# Specific inhibition of NLRP3 inflammasome by a Smurf1 inhibitor *in vitro* and *in vivo*

**DOI:** 10.1515/med-2026-1397

**Published:** 2026-04-09

**Authors:** Lifeng Meng, Hongmei Xuan, Ping Zhou, Weifeng Xu, Wei Bian, Yunxiao Lin, Ying Guan

**Affiliations:** Department of Rheumatology, Zhuji People’s Hospital of Zhejiang Province, Zhuji, China; Department of Pain, Zhuji People’s Hospital of Zhejiang Province, Zhuji, China; Department of Nephrology, Zhuji People’s Hospital of Zhejiang Province, Zhuji, China

**Keywords:** Smurf1, A01, NLRP3 inflammasome, ASC, peritonitis, HFD

## Abstract

**Objectives:**

Abnormal activation of the NLRP3 inflammasome is associated with various inflammatory diseases, making it a potential therapeutic target. A01 is an inhibitor of Smurf1, its effect on the activation of NLRP3 inflammasome is still unclear and needs further study.

**Material and methods:**

Caspase-1 and IL-1β were detected by immunoblotting and ELISA, and (lactate dehydrogenase) LDH release was measured via a specific kit. Immunoprecipitation was used to explore the interaction between NLRP3 and ASC. In a mouse model of alum-induced peritonitis, PECs were collected and analyzed by flow cytometry. In the high-fat diet (HFD) model, blood glucose was measured with a glucometer.

**Results:**

A01 inhibited the activation of the NLRP3 inflammasome in macrophages without affecting the activation of AIM2 or NLRC4 inflammasomes. Mechanistically, A01 suppressed NLRP3 inflammasome assembly and activation by disrupting the NLRP3-ASC interaction. Moreover, A01 demonstrated protective effects in mouse models of NLRP3 inflammasome-mediated diseases.

**Conclusions:**

A01 specifically suppresses NLRP3 inflammasome activation *in vitro* and *in vivo*. A01 disrupts the association of NLRP3 and ASC. These findings suggest that A01 is a specific NLRP3 inhibitor and may be a promising candidate for treating NLRP3 inflammasome-related diseases.

## Introduction

Inflammasomes are cytosolic multiprotein complexes that play a central role in innate immunity by sensing a wide range of microbial and sterile stimuli [1], 2]. Upon activation, inflammasomes promote the cleavage of pro–caspase-1 into its active form, leading to the maturation and secretion of the proinflammatory cytokines interleukin-1β (IL-1β) and IL-18, and to a lytic form of programmed cell death termed pyroptosis [3], 4]. These processes are crucial for host defense but can also drive chronic inflammation and tissue injury when dysregulated [5], [6], [7]. Among the various inflammasome sensors, members of the nucleotide-binding domain and leucine-rich repeat (NLR) family – including NLRP1, NLRP3, and NLRC4 – as well as AIM2 from the PYHIN family, have been well characterized [8], [9], [10]. The NLRP3 inflammasome is the most extensively studied, as it can be activated by diverse pathogen-associated molecular patterns (PAMPs) and damage-associated molecular patterns (DAMPs), such as ATP, nigericin, or crystalline and metabolic danger signals. Activation of NLRP3 triggers the recruitment of the adaptor protein ASC and pro–caspase-1, leading to caspase-1 activation and cytokine release [11], 12]. Persistent or excessive NLRP3 inflammasome activation has been implicated in the pathogenesis of numerous inflammatory and metabolic disorders, including gout, atherosclerosis, type 2 diabetes, nonalcoholic steatohepatitis (NASH), and autoimmune diseases [13], [14], [15], [16], [17]. Thus, pharmacological inhibition of NLRP3 represents an attractive therapeutic strategy, and multiple small-molecule inhibitors targeting NLRP3 or its signaling components have shown beneficial effects in experimental disease models [18], [19], [20], [21], [22]. However, the molecular mechanisms regulating NLRP3 activation remain incompletely understood, and new regulatory pathways or inhibitors are still being identified. Smad ubiquitin regulatory factor 1 (Smurf1) is a HECT-type E3 ubiquitin ligase involved in the regulation of diverse cellular processes, including TGF-β signaling, cell migration, and innate immune responses [23], [24], [25], [26], [27]. A01 is a reported inhibitor of Smurf1, yet its potential involvement in inflammasome regulation has not been investigated.

## Materials and methods

### Antibodies and chemicals

Nigericin (HY-127019) was purchased from MedChemExpress. ATP, flagellin and poly (dA:dT) were purchased from InvivoGen. Ultrapure lipopolysaccharide (LPS), Phorbol 12-myristate 13-acetate (PMA, P8139) and DMSO (D2650) were purchased from Sigma Aldrich. A01 was purchased from Selleck. Anti-mouse caspase-1 (1:1000, AG-20B-0042) and anti-NLRP3 (1:2000, AG-20B-0014) were purchased from Adipogen. Anti-human caspase-1 (1:2000, 4199S) was purchased from Cell Signaling Technology. Anti-ASC (1:1000, sc-22514-R) were purchased from Santa Cruz. Anti-HA (ab9110) was purchased from Abcam. Anti-Flag (20543-1-AP) was purchased from Proteintech.

### Cell culture

Bone marrow-derived macrophages (BMDMs) were isolated from C57BL/6 mice, and cultured in DMEM medium with 10 % FBS, 1 % penicillin/streptomycin (P/S) and 50 ng/mL mouse macrophage colony-stimulating factor (M-CSF) for 6–7 days.

293T cells were cultured in DMEM medium with 10 % FBS, 1 % penicillin/streptomycin (P/S).

THP-1 cells were cultured in RPMI 1640 medium with 10 % FBS, 1 % penicillin/streptomycin (P/S).

The *in vitro* experiment in different cells in this study was repeated three times.

### Cell stimulation

BMDMs were plated at 10^6^/well overnight. The cells were primed with 100 ng/mL LPS for 4 h, then A01 (10 µM or 20 µM) was added 1 h before being stimulated with inflammasome agonists: ATP (5 mM, 1 h), nigericin (10 µM, 30 min), Alum (300 ug/mL, 3 h), poly (dA:dT) (5 μg/mL, 6 h) and flagellin (1 μg/mL, 6 h) [28]. THP-1 cells were plated at 10^5^/well overnight. For siRNA-mediated knockdown of gene expression in BMDMs, 5 pmoles non-targeting (control) siRNA (D-001206-14-20, Horizon Discovery) or Smurf1 siRNA (M-040947-01-0005, Horizon Discovery) per million cells was used for transfection.

### Measurement of cytokine production

Cell culture supernatants were collected during the experiments and the concentrations of.

IL-1β was measured by ELISA kits (Invitrogen, Cat # 88-7013A-88) according to the manufacturer’s instructions.

### Lactate dehydrogenase (LDH) assay

The LDH level in the cell culture supernatants was measured by LDH Cytotoxicity Assay Kit (Promega, Cat # J2380) following the manufacturer’s instructions.

### Immunoblot analysis

The cells were lysed with cell lysis buffer (Cell Signaling Technology) containing protease and phosphatase inhibitors (Sigma-Aldrich) and subjected to SDS-polyacrylamide gel electrophoresis (SDS-PAGE) (Bio-Rad). The cell culture supernatants were precipitated by the addition of an equal volume of methanol and 0.25 vol of chloroform, following by vortexed and centrifuged for 10 min at 15,000 g. The upper phase was discarded, and 500 mL methanol was added to the interphase. This mixture was centrifuged for 10 min at 15,000 g and the protein pellet was dried at 55 °C and subjected to SDS-polyacrylamide gel electrophoresis (SDS-PAGE) (Bio-Rad). After SDS-PAGE, the samples were transferred to a nitrocellulose membrane (Bio-Rad), blocked with 5 % nonfat milk, and incubated with caspase-1 (AG-20B-0042, AdipoGen, 1:1000) overnight. Membranes were then washed and probed with the appropriate horse-radish peroxidase (HRP)-conjugated secondary antibodies (anti-mouse [#315-035-047], 1:5000, Jackson ImmunoResearch Laboratories) for 1 h. Enhanced chemiluminescence was used to visualize the proteins.

### Immunofluorescence

BMDMs were stimulated with ATP, fixed with 4 % paraformaldehyde (PFA) for 10 min, permeabilized with 1 % Triton X-100 for 10 min, and then blocked with 5 % bovine serum albumin (BSA) for 1 h. Then incubated with an ASC primary antibody (Santa Cruz), followed by incubation with appropriate secondary antibodies. Nuclei were counterstained with DAPI.

### Immunoprecipitation

HEK-293T cells were transfected with ASC, NLRP3 plasmids for 24 h, then the cells were incubated with 10 µM A01 for 6 h. Subsequently, samples were lysed with IP lysis buffer (Thermo) and centrifuged at 4 °C, 12,000 g for 15 min, supernatants were then incubated with anti-Flag M2 beads (A2220, MilporeSigma) for 4 h at 4 °C. For BMDM cells, the cells were primed with LPS for 4 h, then A01 (10 µM) was added 1 h before being stimulated with ATP (5 mM, 1 h). The samples were then lysed with IP lysis buffer (Thermo) and centrifuged at 4 °C, 12,000 g for 15 min, supernatants were then incubated with or anti-ASC antibody and protein G-agarose beads overnight at 4 °C. After washing with lysis buffer, the immunocomplexes were dissociated by boiling in loading buffer and subjected to immunoblot analysis.

### Mice

C57BL/6 mice (6–8 weeks) were obtained from the Model Animal Research Center of Nanjing University. Mice were housed in a standard, pathogen-free animal facility under a 12-h light/dark cycle at 22–24 °C with unrestricted access to food and water for the duration of the experiment except during fasting tests (no more than 16 h). Animal experiments were conducted by protocols approved by the Animal Care and Use Committee of Zhuji People’s Hospital of Zhejiang Province Hospital (2023-0106).

### *In vivo* peritonitis

C57BL/6J mice were injected i.p. with 20 mg/kg A01 or vehicle 1 h before i.p. injection of 700 mg alum. Mice were sacrificed 12 h after alum injection and peritoneal cavities were washed with 6 mL of PBS. PECs were collected and analyzed by flow cytometry [29]. IL-1β production in peritoneal lavage fluid was measured by ELISA. The experiment was repeated three times, and there were 6 mice in each group.

### High-fat diet (HFD) model

C57BL/6J mice with similar plasma glucose levels and body weights, were randomized into different groups. For generation of HFD-induced diabetic mice, mice were fed with HFD for 12 weeks. The diabetic mice were treated with A01 (i.p.) at a dose of 3 mg/kg once a day for 4 weeks and maintained with HFD. The experiment was repeated three times, and there were 6 mice in each group.

### Glucose tolerance and insulin tolerance tests

Glucose tolerance tests (GTT) were performed via i.p. injection of glucose (1.5 g/kg) after 14 h fasting from the beginning of the dark cycle. Insulin tolerance tests (ITT) were performed via i.p. injection of human recombinant insulin (1 U/kg) after 4 h fasting. Blood glucose levels were measured from the tail veil at 0, 30, 60, 90, and 120 min after glucose or insulin injection.

### Statistics

GraphPad Prism 9 was utilized for statistical analysis. For the comparison of two groups, unpaired Student’s *t*-test was used. All of the experimental data were presented as mean ± SEM. The difference was considered statistically significant at **p<0.01; ***p<0.001; ns: not significant.

### Ethical approval

The study is approved by Zhuji People’s Hospital of Zhejiang Province, 311800, China (number 2023-0106).

## Results

### A01 potently suppresses the activation of NLRP3 inflammasome

**Figure 1:**
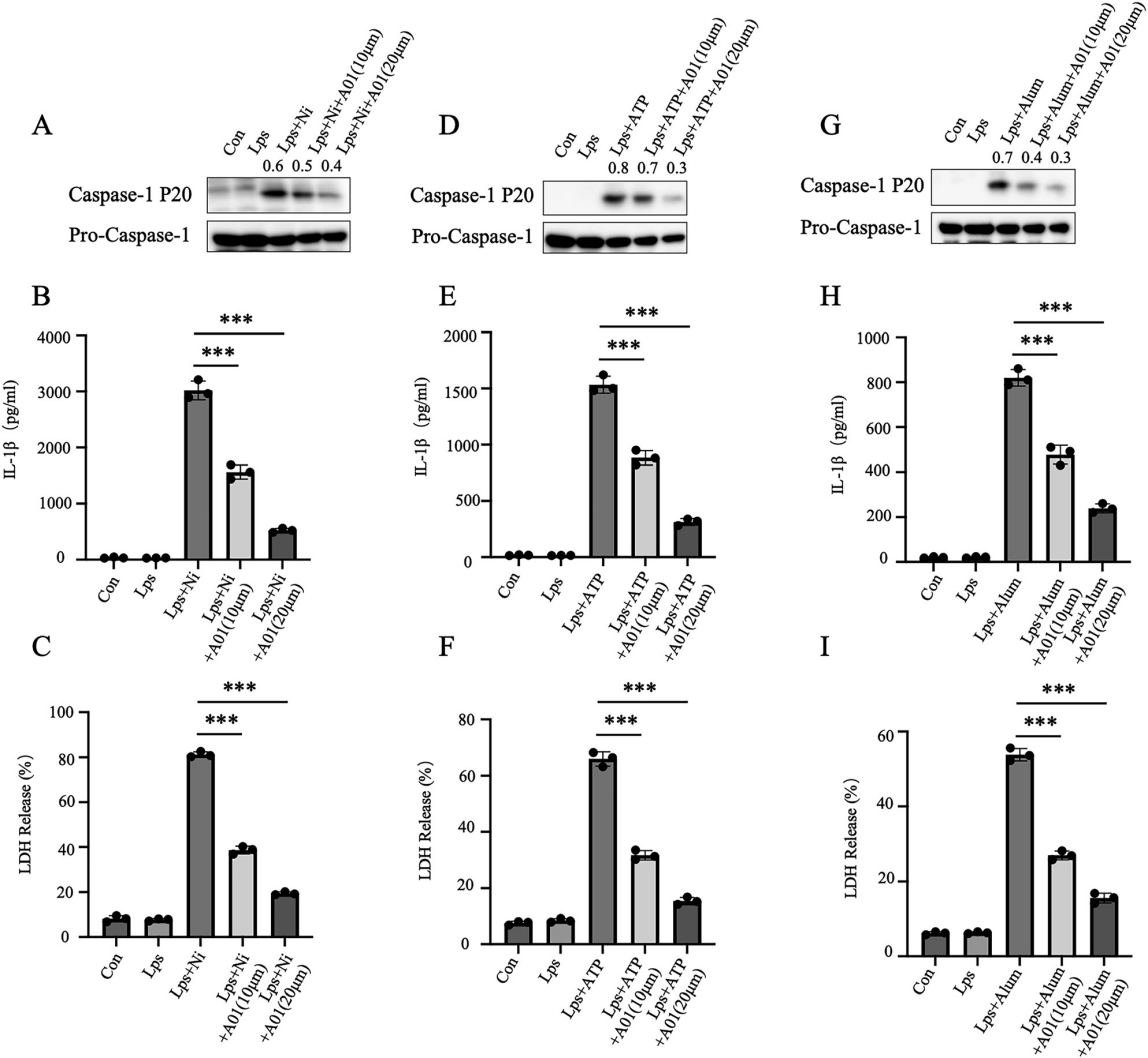
A01 suppressed NLRP3 inflammasome activation in BMDMs. (A–I) Bone marrow–derived macrophages (BMDMs) (100 ng/mL, 4 h) were primed with LPS, followed by incubation with A01 and stimulation with nigericin (10 µM, 30 min) (A–C), ATP (5 mM, 1 h) (D–F), or alum (300 μg/mL, 3 h) (G–I). Cleaved caspase-1 (p20) in the supernatant (Sup) and pro–caspase-1 in cell lysates (Lys) were examined by immunoblotting (A, D, G). IL-1β secretion in the supernatant was quantified by ELISA (B, E, H), and LDH release was measured to assess cell death (C, F, I). Numbers above the blots indicate densitometric ratios of cleaved caspase-1 (p20) to pro–caspase-1. Data represent mean ± SEM of triplicate samples and are representative of at least three independent experiments. ***p<0.001, compared with the LPS + nigericin/ATP/alum group (unpaired Student’s *t*-test).

The activation of the NLRP3 inflammasome triggers caspase-1 cleavage, IL-1β maturation and secretion, (lactate dehydrogenase) LDH release, and pyroptotic cell death [1]. To investigate the direct effect of A01 on inflammasome activation, we employed bone marrow-derived macrophages (BMDMs) as a physiologically relevant *in vitro* model, since they faithfully recapitulate canonical inflammasome activation, including NLRP3, AIM2, and NLRC4 pathways. Bone-marrow-derived macrophages (BMDMs) were primed with LPS and added A01 an hour before stimulated with ATP, nigericin and alum to induce NLRP3 inflammasome activation [28]. Treatment with A01 dose-dependently inhibited caspase-1 cleavage (Figure 1A), IL-1β secretion (Figure 1B) and LDH (Figure 1C) release, induced by nigericin in a dose-dependent manner. The same results were also found in ATP (Figure 1D–F) and alum (Figure 1G–I) -induced NLRP3 inflammasome activation. Furthermore, we found that Smurf1 siRNA-treated BMDMs inhibits NLRP3 inflammasome activation (Figure S1A). The A01 treatment also had effects on NLRP3 activation in THP-1 cells (Figure S1B). Moreover, we found that A01 provided comparable protection against NLRP3 inflammasome activation to the known NLRP3 inhibitor MCC950 (Figure S1C). Collectively, these results demonstrate that A01 effectively suppresses NLRP3 inflammasome activation in BMDMs.

### A01 had no impact on the activation of AIM2 and NLRC4 inflammasome

**Figure 2:**
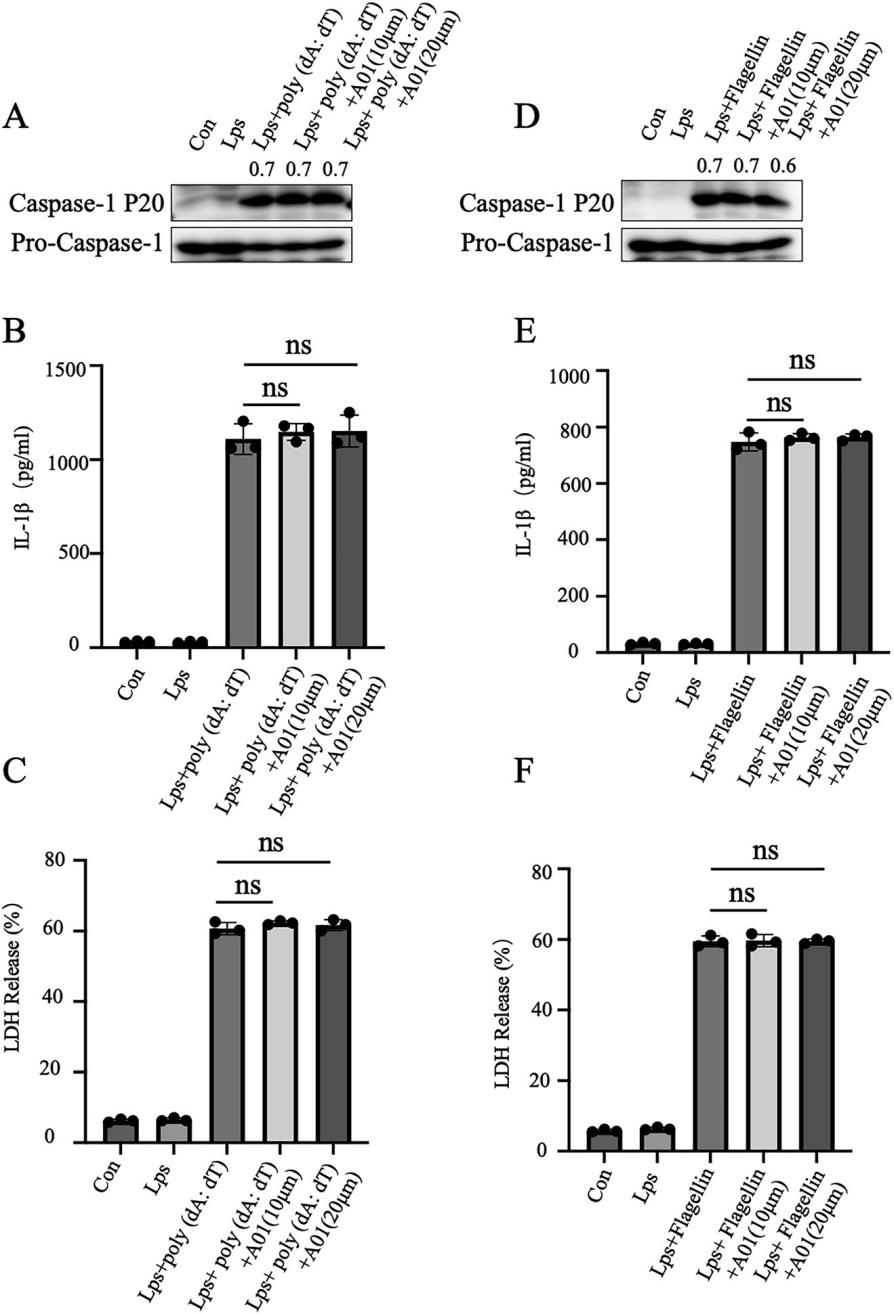
A01 had no impact on NLRC4 or AIM2 inflammasome activation. (A–F) LPS-primed BMDMs (100 ng/mL, 4 h) were treated with or without A01 and then transfected with poly (dA:dT) (5 μg/mL, 6 h) to activate AIM2 (A–C) or flagellin (1 μg/mL, 6 h) to activate NLRC4 (D–F). Cleaved caspase-1 (p20) in the supernatant and pro–caspase-1 in cell lysates were detected by immunoblotting (A, D). IL-1β secretion was measured by ELISA (B, E), and LDH release was assessed (C, F). Numbers above the blots indicate densitometric ratios of cleaved caspase-1 to pro–caspase-1. Data are presented as mean ± SEM of triplicates and represent at least three independent experiments.ns, not significant, compared with the LPS + poly (dA:dT) or LPS + flagellin group (unpaired Student’s *t*-test).

We next studied A01’s impact on the activation of other inflammasomes such as AIM2 and NLRC4, which also mediate caspase-1 activation [30]. After priming, BMDMs were transfected with poly (dA:dT) or flagellin to activate AIM2 or NLRC4 inflammasome activation, respectively [28]. The results showed that A01 inhibited neither caspase-1 cleavage (Figure 2A and D) and IL-1β maturation (Figure 2B and E) nor LDH release (Figure 2C and F). These data confirmed that A01 specifically suppresses the activation of NLRP3 inflammasome, but not AIM2 or NLRC4 inflammasome.

### A01 suppressed ASC oligomerization and restrained NLRP3 inflammasome activation by disrupting NLRP3-ASC interaction

We next explored the mechanism underlying A01’s effect on NLRP3 inflammasome. ASC (Apoptosis-associated speck-like protein containing a CARD) oligomerization plays an important role in caspase-1 cleavage during inflammasome activation [31].

**Figure 3:**
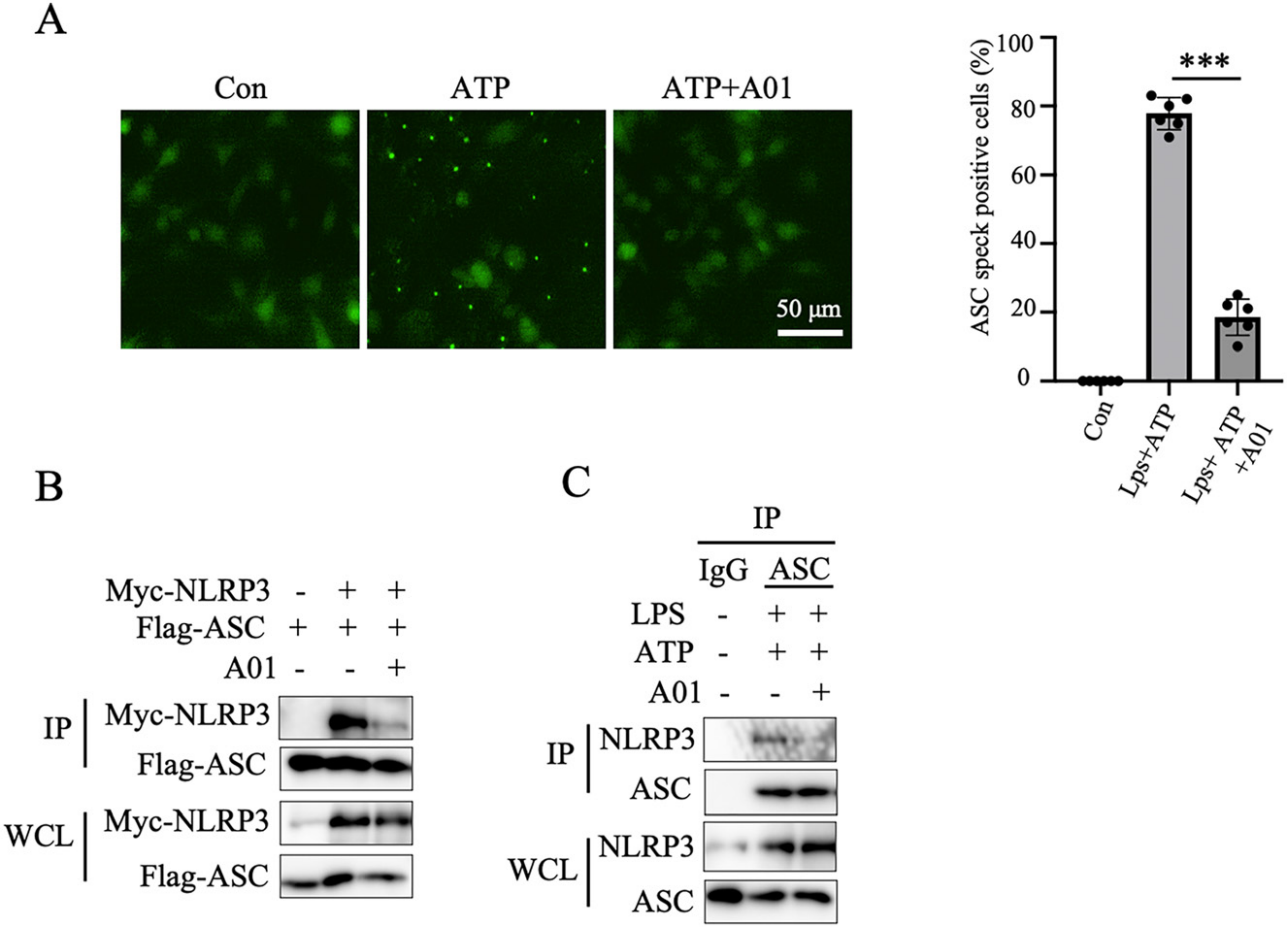
A01 suppressed ASC oligomerization and restrained NLRP3-ASC interaction (A) representative immunofluorescence images showing ASC speck formation in LPS-primed BMDMs (100 ng/mL, 4 h) stimulated with ATP (5 mM, 1 h) in the presence or absence of A01. Scale bar, 50 µm, left; the calculation of ASC speck positive cells, right. (B) Co-immunoprecipitation (Co-IP) analysis of NLRP3–ASC interaction in 293T cells transfected with Myc-NLRP3 and Flag-ASC, treated with or without A01. (C) Co-IP analysis of endogenous NLRP3–ASC interaction in LPS-primed BMDMs treated with A01 followed by ATP stimulation. Data are presented as mean ± SEM and are representative of at least three independent experiments. ***p<0.001, compared with the LPS + ATP group (unpaired Student’s *t*-test).

We found that A01 suppressed the ASC speck formation induced by ATP (Figure 3A). Moreover, investigations were conducted into A01’s impact on NLRP3 inflammasome assembly. We studied whether A01 could prevent the interaction between ASC and NLRP3. Myc-NLRP3 and Flag-ASC were co-overexpressed in 293T cells and then treated with A01. The results showed that A01 treatment inhibited the interaction between ASC and NLRP3 in 293T cells (Figure 3B). We further confirmed this result with ASC antibody in BMDMs (Figure 3C). Thus, our results revealed that A01 suppressed the activation of NLRP3 inflammasome via blocking NLRP3-ASC interaction.

### A01 inhibits alum-induced peritoneal inflammation

**Figure 4:**
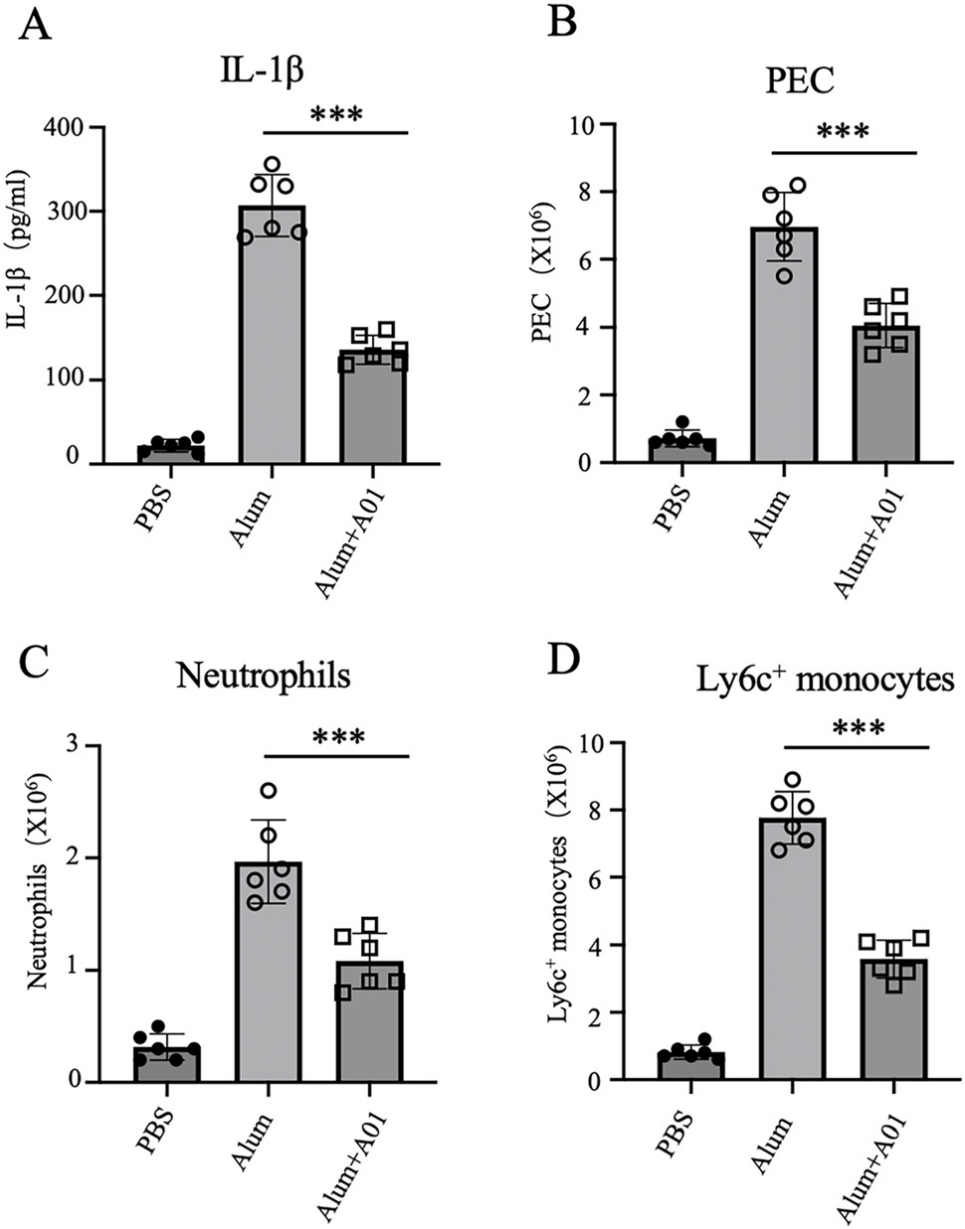
A01 inhibits alum-induced peritoneal inflammation. (A) IL-1β levels in peritoneal lavage fluid collected 12 h after alum (500 µg per mouse) injection with A01 or PBS administration (20 mg/kg, i.p.) (n=6 mice per group). (B–D) Flow cytometric analysis of peritoneal exudate cells (PECs), quantifying total PECs (B), neutrophils (C), and Ly6C^+^ monocytes (D). Data are presented as mean ± SEM and are representative of two independent experiments. ***p<0.001, compared with the alum + A01 treatment group vs. the alum-alone group (unpaired Student’s *t*-test).

To evaluate the *in vivo* relevance of our findings, we employed alum-induced peritonitis, a well-established NLRP3-dependent acute inflammation model. Mice were pretreated with A01 before intraperitoneal (i.p.) injection of alum to induce peritonitis [32].Alum challenge markedly increased mature IL-1β levels in the peritoneal lavage fluid, which were significantly attenuated by A01 pretreatment (Figure 4A). We next assessed the recruitment of inflammatory cells to the peritoneal cavity. Alum induced a substantial increase in total peritoneal exudate cells (PECs), as well as in neutrophils and Ly6C^+^ monocytes, all of which were significantly reduced by A01 (Figure 4B–D). These results indicate that A01 effectively suppresses NLRP3 inflammasome activation and limits subsequent immune cell accumulation *in vivo*.

### A01 ameliorated high-fat diet-induced insulin resistance

**Figure 5:**
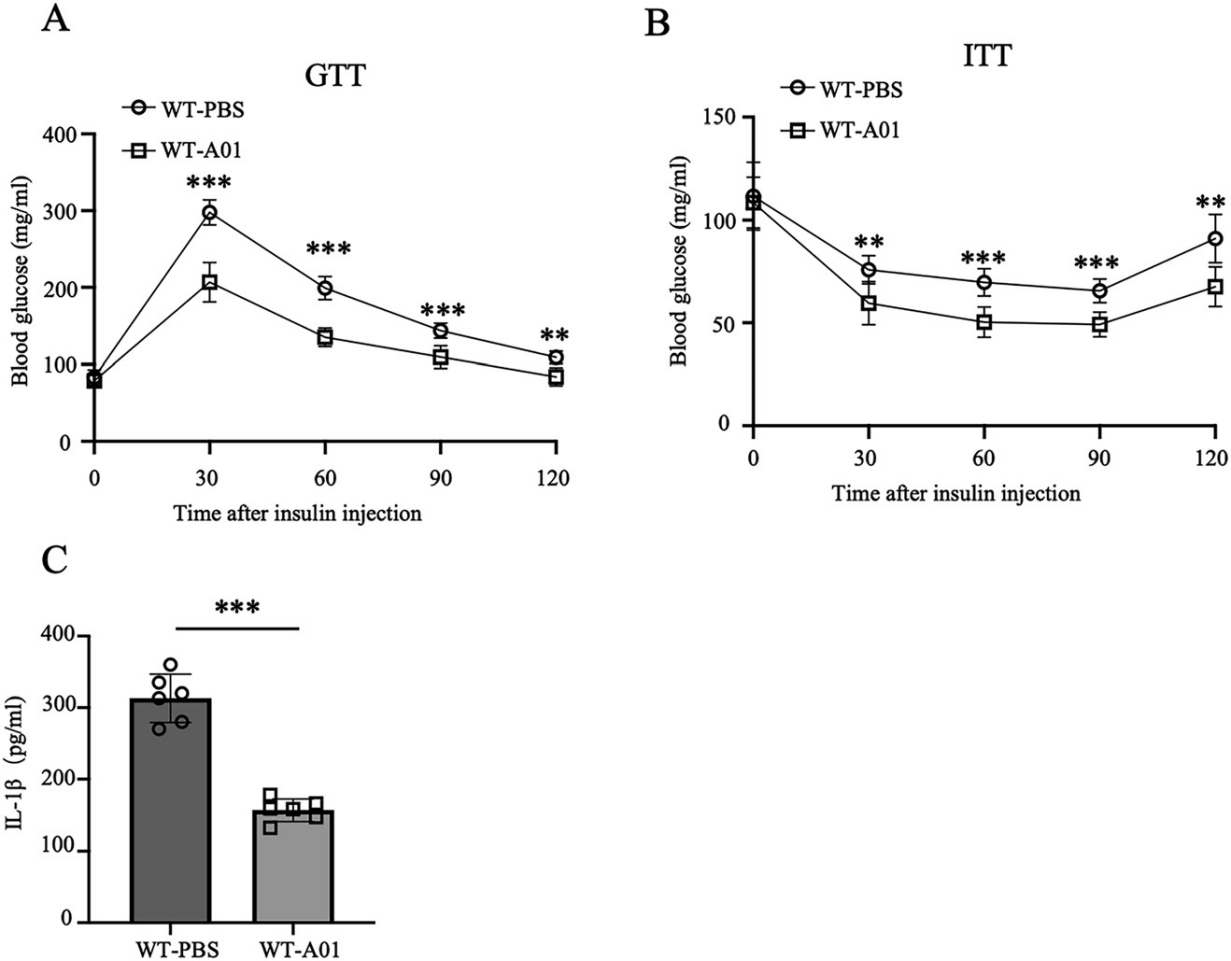
A01 ameliorated high-fat diet-induced insulin resistance mice were fed a high-fat diet (HFD) for 12 weeks and then treated intraperitoneally with A01 (3 mg/kg/day) or PBS for 4 weeks while maintained on HFD (n=6 mice per group). (A, B) glucose tolerance test (A) and insulin tolerance test (B) results. (C) Serum IL-1β concentrations measured by ELISA. Data are presented as mean ± SEM and are representative of two independent experiments. **p<0.01, ***p<0.001, compared with the PBS-treated HFD group (unpaired Student’s *t*-test).

**Figure 6:**
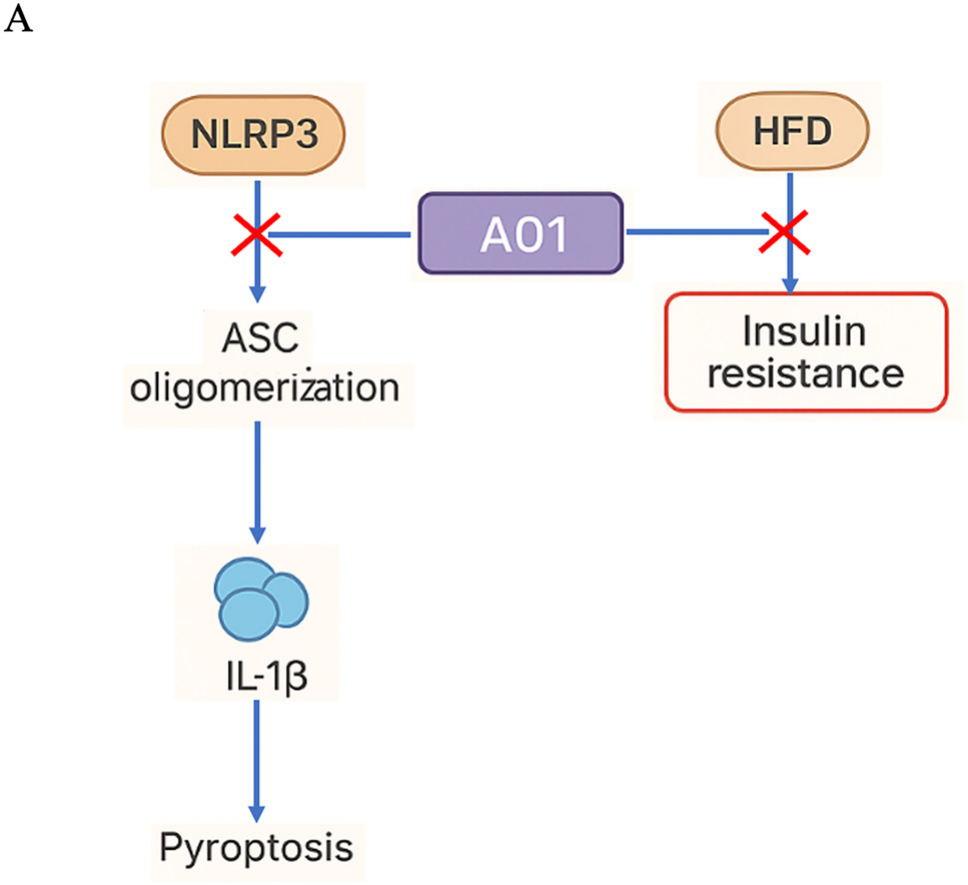
Molecular mechanism of A01-mediated inhibition of NLRP3 inflammasome activation. (A) Molecular mechanism of A01 in NLRP3 inflammasome activation.

We also employed a high-fat diet (HFD)-induced insulin resistance mouse model to investigate the role of A01 in chronic metabolic inflammation, which is closely linked to NLRP3 activation [33], [34], [35]. This model allowed us to assess the protective effects of A01 on glucose tolerance, insulin sensitivity, and systemic IL-1β levels. Consistent with previous evidence that NLRP3-mediated chronic inflammation contributes to insulin resistance and type 2 diabetes (T2D), A01 treatment significantly improved glucose tolerance and insulin sensitivity in HFD-fed mice (Figure 5A and B). Furthermore, ELISA analysis revealed that A01 markedly suppressed IL-1β secretion in serum (Figure 5C). Collectively, these results demonstrate that A01 exerts protective effects against chronic metabolic inflammation *in vivo* (Figure 6).

## Discussion

In this study, we demonstrate that A01 specifically inhibits NLRP3 – but not NLRC4 or AIM2 – activation by disrupting the NLRP3–ASC interaction and preventing inflammasome assembly. Inflammasomes play a critical role in the host response to a wide range of damage- and pathogen-associated molecular patterns by mediating caspase-1 cleavage and subsequent IL-1β processing [36], [37], [38]. Among them, the NLRP3 inflammasome has been implicated in the pathogenesis of numerous inflammatory and metabolic diseases, including NASH, acute lung injury, gout, insulin resistance, and type 2 diabetes [39], [40], [41], [42]. Consequently, tight regulation of NLRP3 activity is essential, and pharmacological targeting of this inflammasome represents a promising therapeutic strategy [4]. Pathogens have evolved strategies to block inflammasome-mediated host defenses, such as the poxvirus protein M013, which binds ASC and inhibits inflammasome activation [3]. Moreover, given the diversity of upstream stimuli and downstream pathological consequences in different disease contexts, a broader repertoire of inhibitors with distinct mechanisms is needed to enable precise, disease-specific interventions [43].

In this study, we demonstrate that A01 is a potent and selective inhibitor of the NLRP3 inflammasome both *in vitro* and *in vivo*. A01 significantly suppressed hallmark features of NLRP3 activation, including caspase-1 cleavage, IL-1β secretion, and pyroptosis-associated LDH release, in response to canonical stimuli such as ATP, nigericin, and alum. Importantly, A01 exhibited no detectable effect on the activation of AIM2 and NLRC4 inflammasomes, underscoring its specificity for the NLRP3 pathway. Mechanistically, we showed that A01 interferes with NLRP3 inflammasome assembly by blocking the interaction between NLRP3 and ASC, and thereby inhibits ASC oligomerization. Since ASC oligomerization is essential for caspase-1 activation and subsequent inflammatory cytokine maturation [3], disruption of this process by A01 represents a crucial molecular mechanism underlying its inhibitory function. These results are consistent with previous studies suggesting that small-molecule inhibition of NLRP3-ASC interaction represents a viable strategy for therapeutic targeting of inflammasome-driven diseases [44], 45].

Our *in vivo* findings further support the therapeutic potential of A01. In a mouse model of alum-induced peritonitis, A01 treatment significantly reduced IL-1β secretion and inflammatory cell recruitment, including neutrophils and Ly6C^+^ monocytes. These findings highlight the capacity of A01 to dampen sterile inflammation *in vivo*, an important feature for potential clinical translation. Moreover, we observed that A01 ameliorates insulin resistance in a high-fat diet-induced mouse model. As chronic low-grade inflammation mediated by the NLRP3 inflammasome contributes to metabolic disorders such as type 2 diabetes [35], our data suggest that A01 may serve as a promising candidate for managing obesity-associated metabolic inflammation. The observed reduction of systemic IL-1β levels following A01 treatment further supports its systemic anti-inflammatory activity. Recent studies have introduced innovative approaches to investigate inflammasome activation and its therapeutic modulation. For instance, newly developed chemical probes and genetic tools have enabled more precise assessment of NLRP3 dynamics and downstream signaling in inflammatory diseases [46], [47], [48]. Incorporating these advanced methods in future studies will help to further define the molecular targets and *in vivo* pharmacological mechanisms of A01, thereby strengthening its translational potential for metabolic and inflammatory disorders.

Despite these encouraging findings, our study has several limitations. First, while BMDMs provide a physiologically relevant *in vitro* system, they may not fully capture the complexity of human immune responses. Second, the *in vivo* studies were limited to two mouse models, and additional models of NLRP3-driven diseases are needed to generalize the therapeutic potential of A01. Third, the pharmacokinetics, bioavailability, and long-term safety of A01 remain to be evaluated, which are critical considerations for potential clinical translation. Finally, although A01 selectively inhibits NLRP3 in our assays, off-target effects in other cell types or under different inflammatory contexts cannot be completely excluded.

Future studies should address these limitations. Further preclinical investigations are warranted to assess A01 in diverse models of NLRP3-mediated diseases, including autoimmune disorders and chronic inflammatory conditions. Detailed pharmacological profiling and structure-activity relationship studies may optimize its potency, selectivity, and safety. In addition, exploring combination therapies with existing anti-inflammatory agents could reveal synergistic benefits. Mechanistic studies could also determine whether A01 modulates upstream signaling pathways or interacts with other inflammasome regulators, thereby broadening our understanding of its mode of action. our data suggest that A01 disrupts the NLRP3–ASC interaction, further studies in primary cells and *in vivo* models are required to confirm this mechanism in physiological contexts. Although A01 selectively inhibits NLRP3 inflammasome activation, its effects on upstream regulators such as NEK7 or potassium efflux remain to be determined. The contribution of Smurf1 vs. potential off-target effects also requires further study using knockdown or rescue experiments. In addition, our results are limited to murine models, and the pharmacokinetics, safety, and translational potential of A01 need to be evaluated in future preclinical studies. Compared with established inhibitors such as MCC950, A01 shows promising activity, but additional work is necessary to fully assess its clinical applicability.

In summary, our results identify A01 as a selective and effective NLRP3 inflammasome inhibitor that functions by disrupting NLRP3-ASC interactions. These findings provide a foundation for future preclinical development and suggest that A01 holds potential as a therapeutic agent for NLRP3-related inflammatory and metabolic diseases.

## Supplementary Material

Supplementary Material

Supplementary Material
