# Supplementary material for: Specific inhibition of NLRP3 inflammasome by a Smurf1 inhibitor in vitro and in vivo
Source: Open Med (Wars). 2026 Apr 9;21(1):20261397. doi: 10.1515/med-2026-1397 (PMC13068877; doi:10.1515/med-2026-1397)
Supplement: Supplementary file 1 — Supplementary Material [file j_med_2026-1397_suppl_001.docx]

Fig. S1 Smurf1 deficiency inhibited NLRP3 inflammasome activation. (A) Bone marrow–derived macrophages (BMDMs) were treated with control or Smurf1 siRNA, followed by primed with LPS and stimulation with nigericin ATP (5 mM, 1h). Data are representative of at least three independent experiments. (B) THP-1 cells were primed with LPS, followed by incubation with A01 and stimulation with ATP (5 mM, 1h). Cleaved caspase-1 (p20) in the supernatant (Sup) and pro–caspase-1 in cell lysates (Lys) were examined by immunoblotting. (C) Bone marrow–derived macrophages (BMDMs)were primed with LPS, followed by incubation with A01(20 µM), or MCC950 (20 µM), and stimulation with ATP (5 mM, 1h). Cleaved caspase-1 (p20) in the supernatant (Sup) and pro–caspase-1 in cell lysates (Lys) were examined by immunoblotting (A, D, G).
