# Supplementary figures and images for: Specific inhibition of NLRP3 inflammasome by a Smurf1 inhibitor in vitro and in vivo
Source: Open Med (Wars). 2026 Apr 9;21(1):20261397. doi: 10.1515/med-2026-1397 (PMC13068877; doi:10.1515/med-2026-1397)

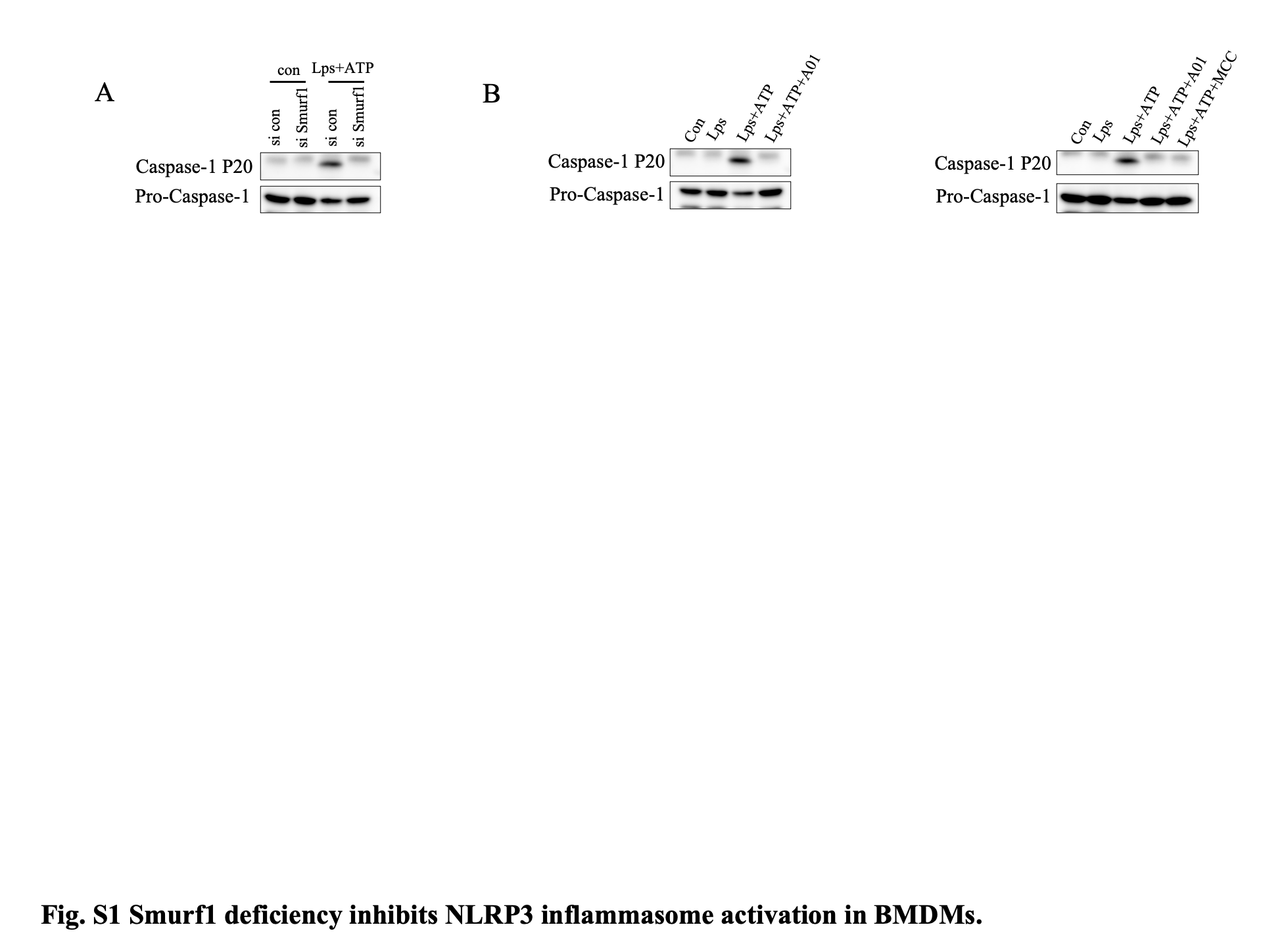

Supplement: Supplementary file 2 — Supplementary Material [file j_med_2026-1397_suppl_002.png]
